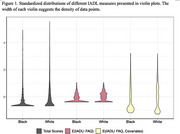# Beyond Total Scores: Investigating Bias in Functional Assessment Questionnaire Ratings Using a Multivariate Differential Item Functioning Approach

**DOI:** 10.1002/alz70857_105319

**Published:** 2025-12-25

**Authors:** Zeling He, John Hanfelt, Felicia C. Goldstein

**Affiliations:** ^1^ Emory University Rollins School of Public Health, Atlanta, GA, USA; ^2^ Emory University School of Medicine, Atlanta, GA, USA

## Abstract

**Background:**

Instrumental activities of daily living (IADLs) are used to diagnose older adults as having mild cognitive impairment (MCI) versus dementia. IADL rating scales, however, can be subject to biases. These biases, known as differential item functioning (DIF), occur when individuals receive different ratings based on demographic or contextual factors. Evaluating and addressing DIF is crucial because of the impact of IADL ratings on eligibility for clinical trials and access to FDA approved treatments.

**Method:**

The Functional Activities Questionnaire (FAQ), an informant IADL rating scale, was available for 7,958 participants with MCI in the National Alzheimer's Coordinating Center Uniform Data Set. Data were analyzed using the Likelihood‐based Investigation of Differential Item Functioning (LIDIF) model. This multivariate approach evaluates uniform and non‐uniform DIF while simultaneously adjusting for multiple covariates, including participants’ age, sex, education, race, and informants’ sex and cohabitation status in a multivariate setting. DIF‐adjusted IADL levels were estimated using posterior means to mitigate rating biases

**Result:**

Significant DIF effects were identified across all 10 FAQ items, with race and informant characteristics emerging as key sources of bias. Black participants were consistently rated as less impaired than white participants, particularly at higher impairment levels, even after adjusting for age, education, and other covariates. This was most pronounced for items related to paying bills, shopping, games, meal preparation, paying attention, remembering appointments and travelling. Informants’ cohabitation status also influenced ratings, with non‐cohabitating informants generally reporting less functional impairment compared to cohabitating informants. FAQ total scores underestimated impairment in Black participants due to a strong floor effect, where a large proportion of individuals received the lowest possible scores. DIF‐adjusted IADL estimates reduced racial disparities, mitigated floor effects, and identified a greater number of individuals with impairment.

**Conclusion:**

These findings highlight the importance of accounting for demographic and informant‐related characteristics in FAQ assessment. Adjusting for these biases improves the accuracy and fairness of IADL assessments, supporting equitable clinical decision‐making and ensuring that functional impairment is appropriately recognized across diverse populations.